# Key considerations for a pre-emergency survival pack: a hypothetical case study

**DOI:** 10.1186/2046-7648-4-S1-A63

**Published:** 2015-09-14

**Authors:** Alvin Khah, Jason Lee

**Affiliations:** 1 Human Performance Laboratory, Defence Medical and Environmental Research Institute, DSO National Laboratories. Singapore

## Introduction

Populations affected by natural disasters usually rely heavily on search and rescue operations and relief supplies to sustain their road to recovery. It is observed that few survivors are recovered after 2 weeks^1 ^of continuous search and rescue efforts and limited resources are distracted from aiding rescued survivors. Hence, individuals living in natural disaster prone areas or have received early warning for an impending disaster may consider owning/be provided with a pre-emergency nutritional pack for self-sufficiency. In addition, collapsed structures may entrap victims in confined spaces with limited oxygen supply and faces the danger of hypercapnia, so a hypothetical example of a 20 year old healthy male in 14 day entrapment was used to demonstrate the dietary, CO_2 _output and CO_2 _scrubbing requirements. A commercially available 20 g protein bar and lithium hydroxide (LiOH) powder were used in the calculations to show how the content of such a pre-emergency pack can be tailored.

## Methods

Calculations based on published sources of dietary requirements and CO_2 _emission of an adult.

## Discussion

The dietary requirements in Table 1 provide the minimum nutrition for sustenance and minimise CO_2 _output from the entrapped 20 year old healthy male weighing 70 kg through 14 days in a 10 m^3 ^space. The CO_2 _scrubbing capacity of this pre-emergency pack should also scrub any CO_2 _produced through respiration by the victim during this period of entrapment. Any dietary and/or CO_2 _scrubbing alternatives can be evaluated against these requirements and selected into the pre-emergency pack.

**Table 1:** Emergency pack requirements for a 20 year old healthy male of 70 kg to survive an entrapment in 10 m^3 ^space with healthy weight loss and maintaining CO_2 _concentration at initial level.

	Daily	14 days	Example of pre-Emergency Pack for 14 days
**Energy**	1190 kcal	16660 kcal	**70 bars of "commercially available 20 g protein bar"**

**Carbohydrate **[2]	130 g	1820 g	

**Protein **[2]	56 g	784 g	

**Fat**	49.5 g	693 g	

**Sodium **[2]	500 mg	7000 mg	

**Water **[3]	2.5 L	35 L	**35 L**

**CO_2 _to be scrubbed**	0.637 m^3^	8.918 m^3^	**24 kg of LiOH**

## Conclusion

Prevention is better than cure in most, if not all, harmful situations. This novel pre-emergency survival pack is a convenient "first aid kit" for natural disasters. A carefully designed pre-emergency pack can be easily adapted to the unique conditions of various disasters and enhance the survivability of a victim entrapped under debris at a site of natural disaster. Early distribution of pre-emergency survival packs will also ensure isolated rural dwellers to be self-sufficient, during the aftermath of a natural disaster, while awaiting arrival of relief supplies.
